# Data on resilience and trauma-related symptoms in Lithuanian children victims of violence

**DOI:** 10.1016/j.dib.2019.103791

**Published:** 2019-02-28

**Authors:** F. Giordano, F. Ragnoli, F. Brajda Bruno

**Affiliations:** Department of Psychology – Resilience Research Unit, Università Cattolica del Sacro Cuore Largo Gemelli 1, 20121, Milan, Italy

## Abstract

The current data article presents a dataset about resilience and trauma-related outcomes in a group of Lithuanian children victims of violence, who took part in a resilience-focused therapy - the Assisted Resilience Approach Therapy (ARAT). The Child Youth Resilience Measure (CYRM-28) and the Trauma Symptom Checklist for Children (TSCC) were administered before and at the end of the treatment. Participants were 65 children (mean age = 13.03; range = 9–17) victims of different types of violence and neglect, referred to 25 day-care centers across Lithuania specialized in child violence. A structural equation modelling (SEM) was performed to test direct relationship between the resilience increase over the treatment and the trauma-related outcomes at the end of it, by controlling the direct effect of trauma-related symptoms at the baseline on the outcomes. This data-in-brief article accompanies the paper: “Resilience and trauma-related outcomes in children victims of violence attending the Assisted Resilience Approach Therapy (ARAT). *Children and Youth Services Review.”* [1].

**Table:** Specifications table

Subject area	*Psychology*
More specific subject area	*Developmental Psychology.*
Type of data	*Figure and chart*
How data was acquired	*Individual assessment of patients by psychotherapists.*
Data format	*Raw data files of item-level data and of summary scores.*
Experimental factors	Sample consisted of 65 Lithuanian children victims of violence, referred to 25 day-care centres specialized in child violence, attending a resilience-focused therapy.
Experimental features	Resilience and trauma-related symptoms were assessed before and after the resilience-focused therapy, through the Child Youth Resilience Measure (CYRM-28) and the Trauma Symptom Checklist for Children (TSCC). Statistical analysis was conducted using IBM SPSS Statistics Version 23 and MPLUS Version 7 software.
Data source location	*Lithuania.*
Data accessibility	Data is included in this article
Related research article	Giordano, F., Ragnoli, F., Brajda-Bruno, F., Boerchi, D. Resilience and trauma-related outcomes in children victims of violence attending the Assisted Resilience Approach Therapy (ARAT). *Children and Youth Services Review,* (in press) [1].

**Table:** 

**Value of the data** • This data provides information on resilience and trauma-related outcomes in a group of Lithuanian children victims of different type of violence, before and after attending a resilience-focused psychotherapy (the ARAT). • The relation between resilience and trauma-related symptoms can be compared with other groups of children victims of different types of trauma. • The data was collected in Lithuania and has cultural diversity value.

## 1 Data

The data comprised a dataset about resilience and trauma-related outcomes in a group of 65 Lithuanian children victims of violence, who took part in a resilience-focused therapy - the Assisted Resilience Approach Therapy (ARAT). The sample was assessed before and after the treatment. Resilience is here defined as the process of successful adaptation despite challenging or threatening circumstances [2], [3], [4], [5].

**Fig. 1:**
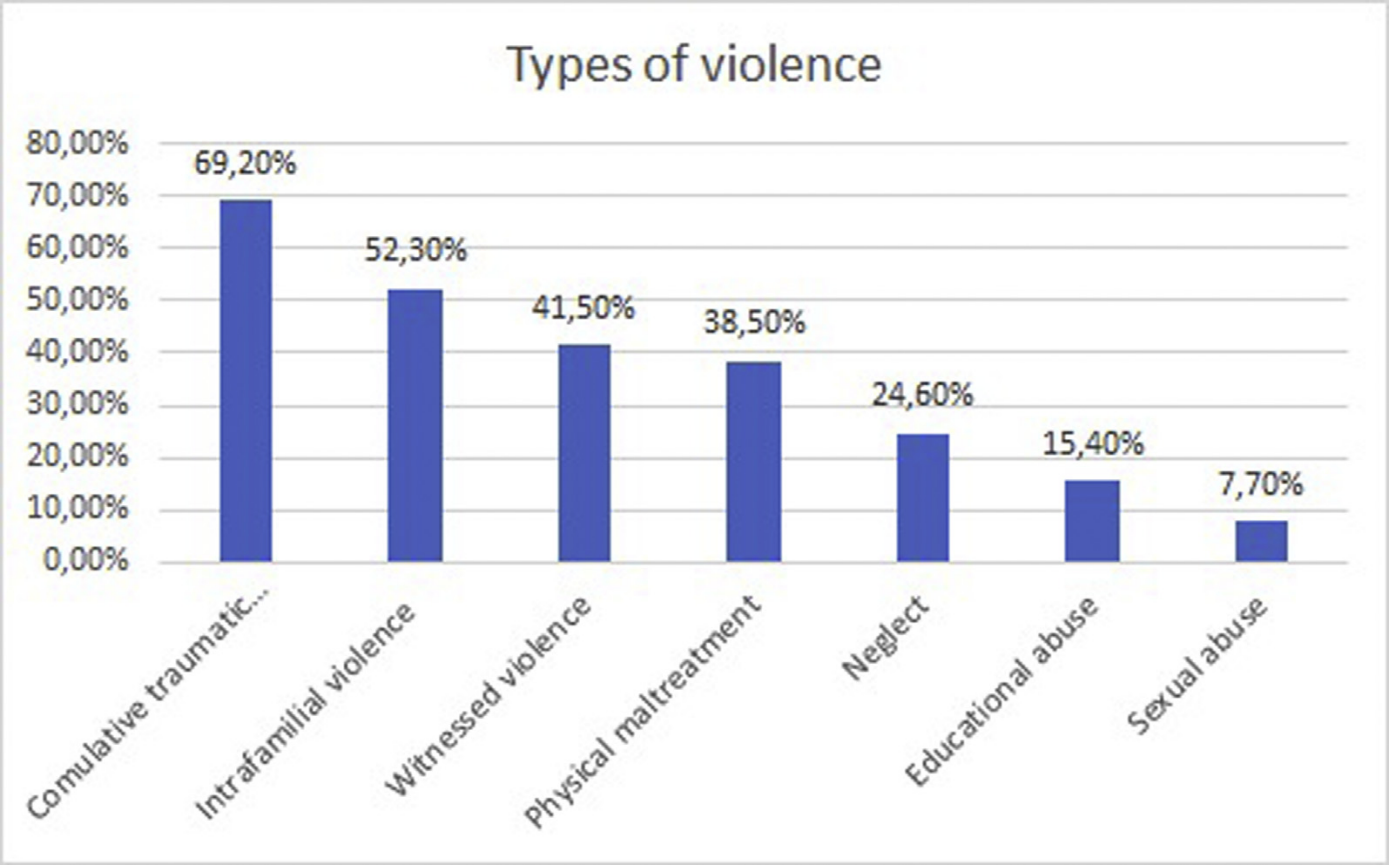
Types of violence.

**Fig. 2:**
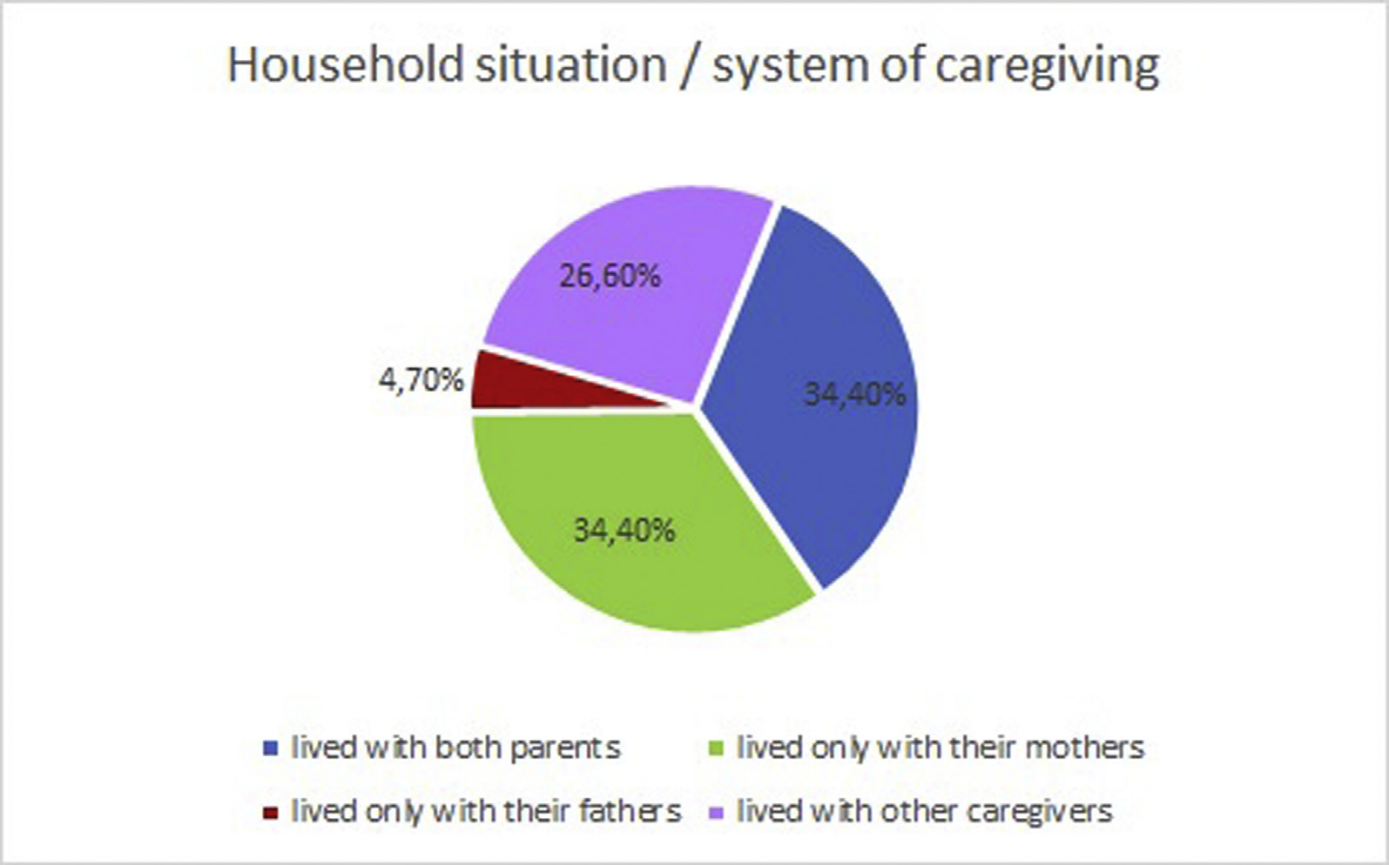
Household situation.

In the following sections, results concerning the demographic and epidemiological variables of the sample are reported. In particular, for the current study we considered qualitative data concerning the specific types of violence the participants were exposed to (Fig. 1), and qualitative data concerning the domestic situation of the participants, namely, with whom they lived (Fig. 2). Furthermore, quantitative data concerning the participants’ levels of trauma-related symptomatology and resilience were assessed through the use of, respectively, the *Trauma Symptom Checklist for Children* and the *Children and Youth Resilience Measure*-*28.*

The goal of the study was to determine whether the increase in resilience along with the treatment would predict the outcomes of trauma-related symptoms scores at the end of the ARAT. As shown in the following sections, statistical analysis subsequently conducted on the data here presented allowed the researches to determine the positive effects of an increase in resilience levels in terms of reduction of anger and dissociation trauma-related symptoms.

## 2 Experimental design, materials, and methods

### 2.1 Participants

The study sample consisted of 65 Lithuanian children (33 boys and 32 girls) with a mean age 13.03 years (SD = 2.11), victims of different types of violence referred to 25 day-care centers specialized in assisting children victims of violence. Children received 22 weekly sessions of the ARAT in the day-care centers between November 2016 and June 2017. No therapy fees were previewed for the participants.

### 2.2 Procedures

The sample recruitment was conducted by the day-care centers’ therapist(s) involved in the ARAT program, and coordinated by the “Paramos Vaikams Centras”, the first center in Lithuania specialized in the assistance of children victims of violence. Participants were either self-referred or referred from child protective services, child advocacy centers, and schools, due to their personal histories of violence. Informed consent was provided by caregivers or other legal guardians for all the participants after an oral presentation offered by the therapist to both child and caregiver about the survey and the pilot treatment. Participants were given the option of declining without penalty. No one chose to decline.

All the therapists involved in the program assisted to the ARAT training run by two psychotherapists of the Resilience Research Unit of the Università Cattolica del Sacro Cuore in September 2016.

Two data collections were conducted along the ARAT implementation (October 2016, at the baseline, and June 2017, at the end, after 22 sessions of the ARAT). The two questionnaires administrations were run by psychotherapists in the clinical setting.

Research procedures were reviewed and approved by the Scientific Committee of the Department of Psychology Resilience Research Unit (RiRes) of the Università Cattolica del Sacro Cuore and followed the standard guidelines for research.

The data were coded and inputted in SPSS Statistics Version 23.

### 2.3 Measures

The *Trauma Symptom Checklist for Children* (TSCC) [6] is a 54-item questionnaire aimed to assess the effects of childhood trauma through the child's self-report of trauma-related symptoms. It is composed by 6 clinical scales: Anxiety, Depression, Posttraumatic Stress, Dissociation, Anger, and Sexual Concerns.

The *Child and Youth Resilience Measure-28 items* (CYRM-28) [7] is a 28 items scale designed to assess factors related to resilient outcomes. For each question, participants used a 3-point Likert scale ranging from 1 (“not at all”) to 3 (“a lot”). The resources are conceptualized and measured as belonging to three subscales: Individual Skills (IS), Relationship with primary Caregiver (RC) and Context Factors (CF). For each subscale of the CYRM-28 there are sub-clusters of questions. The sub-clusters for Individual Skills are Personal Skills, Peer Support, and Social Skills; the ones for Relationship with primary Caregiver are Physical Caregiving and Psychological Caregiving; and the sub-clusters for the Context Factors are Spiritual, Education and Cultural.

The two instruments did not exist in a Lithuanian version; therefore, they have been first independently translated in Lithuanian by a professional translator. This initial translation was submitted to a group of English-speaking psychotherapists participants to the ARAT training. The integrity of the scales was then verified using the back-translation technique [8]. Discrepancies with the original English version were noted, and the Lithuanian version was adjusted accordingly.

### 2.4 Statistical treatment

Wilcoxon Signed Rank test was performed to determine whether the increase of resilience and the decrease of trauma-related symptoms between the baseline and the end of the ARAT were statistically significant. Analysis showed a statistically significant decrease of Anger, Anxiety, Depression, Dissociation, and PTSD, paralleled with the ARAT, and a statistically significant increase of the Child Youth Resilience Measure. Resilience scores presented a statistically significant increase before and after the ARAT except for Caregiver subscale (Fig. 2).

A structural equation modelling (SEM) analytic technique was used to test direct relationship between the scales of TSCC and Δ CYRM, by controlling the direct effect of trauma-related symptoms at the baseline on the outcomes.

This regression analysis revealed some significant coefficients of Resilience Total Score on Anger and Δ Resilience Total Score on Dissociation, Dissociation at baseline (T_0_) on dissociation at the end of the treatment (T_2_); of Anger (T_0_) in Anger (T_2_); of depression (T_0_) on depression (T_2_); of PTSD (T_0_) on PTSD (T_2_); of Anxiety (T_0_) on Anxiety (T_2_).
